# Role of ZnuABC and ZinT in *Escherichia coli *O157:H7 zinc acquisition and interaction with epithelial cells

**DOI:** 10.1186/1471-2180-11-36

**Published:** 2011-02-21

**Authors:** Roberta Gabbianelli, Raffaella Scotti, Serena Ammendola, Patrizia Petrarca, Laura Nicolini, Andrea Battistoni

**Affiliations:** 1 Biotechnology Service of Service for Biotechnology and Animal Welfare, Istituto Superiore di Sanità, Viale Regina Elena 299, 00161 Rome, Italy; 2 Department of Biology, University of Rome "Tor Vergata", Via della Ricerca Scientifica, 00133 Rome, Italy

## Abstract

**Background:**

Zinc is an essential element for all living cells. Recent studies have shown that the ZnuABC zinc uptake system significantly contributes to the ability of several pathogens to multiply in the infected host and cause disease, suggesting that zinc is scarcely available within different tissues of the host. To better understand the role of zinc in bacterial pathogenicity, we have undertaken a functional characterization of the role of the ZnuABC-mediated zinc uptake pathway in enterohemorrhagic *Escherichia coli *O157:H7.

**Results:**

In this work we have analyzed the expression and the role in metal uptake of ZnuA, the periplasmic component of the ZnuABC transporter, and of ZinT, another periplasmic protein which has been shown to contribute to zinc recruitment. We report that the expression of *zin*T and *znu*A, regulated by Zur, is induced in zinc-poor media, and that inactivation of either of the genes significantly decreases *E. coli *O157:H7 ability to grow in zinc depleted media. We also demonstrate that ZinT and ZnuA have not a redundant function in zinc homeostasis, as the role of ZinT is subordinated to the presence of ZnuA. Moreover, we have found that *znu*A and *zin*T are strongly induced in bacteria adhering to cultured epithelial cells and that lack of ZnuA affects the adhesion ability. In addition we have found that a fraction of apo-ZinT can be secreted outside the cell where the protein might sequester environmental zinc, inducing a condition of metal starvation in surrounding cells.

**Conclusions:**

The here reported results demonstrate that ZnuABC plays a critical role in zinc uptake also in *E. coli *O157:H7 and that ZinT contributes to the ZnuA-mediated recruitment of zinc in the periplasmic space. Full functionality of the zinc import apparatus is required to facilitate bacterial adhesion to epithelial cells, indicating that the microbial ability to compete with the host cells for zinc binding is critical to establish successful infections. The observation that ZinT can be secreted when it is in the apo-form suggests that its presence in the extracellular environment may somehow contribute to metal uptake or facilitate bacterial colonization of the intestinal epithelia.

## Background

Transition metals play an essential role in all organisms as they are used as structural or catalytic cofactor in a very large number of proteins [1]. Among these elements, zinc is the one which is found in the largest number of enzymes with known three-dimensional structure [2] and recent bioinformatics investigations have established that zinc-binding proteins constitute about 5% of bacterial proteomes [3]. Despite its abundant employment in proteins, the intracellular concentration of zinc must be accurately controlled to prevent its potential toxicity. To this aim bacteria have developed effective systems to regulate the balance between uptake and export of zinc and maintain an optimal intracellular level of this metal [4-6]. In *Escherichia coli *K12, for example, zinc efflux is achieved through the two transporters ZitB, a member of the cation diffusion facilitator family [7], and ZntA, a P-type ATPase [8]. ZntA synthesis is regulated by ZntR [9], a zinc-responsive Mer-like transcriptional regulator that activates *znt*A transcription by binding to zinc, thus favoring the efflux from the cell of the metal in excess. Zinc uptake is ensured by a few transporters characterized by different affinity for the metal. Under conditions of moderate zinc availability, metal uptake is carried out by the low affinity permease ZupT, a member of the ZIP family of transporters [10]. In contrast, when bacteria grow in environments characterized by very low zinc availability, zinc import is ensured by the high affinity zinc transporter ZnuABC [4,11], whose synthesis is tightly controlled by the binding of this metal to the promoter of *zur *gene [12]. Studies carried out in different bacterial species have established that ZnuABC is strictly required to promote an efficient microbial growth in media deficient in zinc and to ensure bacterial virulence, indicating that zinc availability in the infected host is very limited and that several bacteria strictly rely on this specific transporter to compete with their host for zinc binding [13-20].

It has been recently shown that in some bacterial species the fine-tuning of zinc uptake involves another protein, ZinT (formerly known as YodA), which was initially identified in *E. coli *as a cadmium stress stimulated protein [21-23]. Subsequent investigations have demonstrated that ZinT is involved in periplasmic zinc binding under zinc-limiting conditions [24,25] and it has been hypothesized that it could play a zinc-chaperone role by delivering metal ions to apo-proteins in need of their cofactor [12]. More recently, studies carried out in *Salmonella enterica *serovar Typhimurium have suggested that ZinT participates to the zinc uptake process mediated by ZnuABC, through a mechanism involving its direct interaction with ZnuA [18]. Such a role, however, appears to be dispensable, as many bacteria expressing ZnuABC do not possess ZinT [18].

To strengthen our knowledge on the relevance of zinc import in the host-pathogen interaction, we analyzed the role of ZnuABC and ZinT in the enterohemorrhagic *E. coli *O157:H7 strain. This pathogen is able to colonize the large intestine mucosa of humans, where it causes characteristic attaching and effacing lesions on intestinal epithelial cells which are responsible for the major symptoms of hemorrhagic colitis and Haemolytic Uremic Syndrome (HUS) [26]. Our results highlight the central importance of this zinc uptake pathway in *E. coli *O157:H7 and confirm the participation of ZinT to the mechanisms of metal import mediated by the high affinity zinc transporter ZnuABC.

## Methods

### Reagents

Antibiotics, bovine serum albumin and D-MEM, were purchased from Sigma-Aldrich. Restriction endonuclases, DNA-modifying enzymes and DNA polymerase High-Fidelity Expand were obtained from Roche, while Euro*Taq *and *Pfu *DNA polymerases were obtained from EuroClone and Promega, respectively. All other chemicals were purchased from BDH and were of the highest available grade. The oligonucleotides were synthesized by Primm (Milan, Italy).

### Strains and growth conditions

All strains used in this work are listed in Table 1. *E. coli *O157:H7 ED597 is a clinical human isolate associated to a HUS case [27].

**Table 1:** Bacterial strains

Strains	Relevant genotype or characteristic	Reference or source
*E. coli *O157:H7		
ED597	Wild type	D'Orazio *et al*., 2008
RG112	Δ*zinT*::*kan*	this study
RG113	Δ*znuA*:: *kan*	this study
RG114	Δ*znuA*::*cat *Δ*zin*::*kan*	this study
RG115	Δ*etpC*::*cat*	this study
RG-F116	*zinT*::3xFLAG-*kan*	this study
RG-F117	*znuA*::3xFLAG-*kan*	this study
RG-F118	Δ*zur*::*cat zinT*::3xFLAG-*kan*	this study
RG-F119	Δ*zur*::*cat znuA*:: 3xFLAG-*kan*	this study
RG-F120	Δ*zinT*::*cat znuA*::3xFLAG- *kan*	this study
RG-F121	Δ*znuA*::*cat zinT*::3xFLAG- *kan*	this study
RG-F122	Δ*etpC*::*cat zinT*::3xFLAG- *kan*	this study
RG-F123	Δ*etpD*::*cat zinT*::3xFLAG- *kan*	this study
		
*E. coli*		
BL21	Wild type	laboratory collection
DH 5α	Wild type	laboratory collection
		
*S. enterica*		
PP134	*zinT*::3xFLAG- *kan*	Petrarca *et al*., 2010
SA140	*znuA*::3xFLAG- *kan ilv*I::Tn10dTac- *ca*t:: 3xFLAG- *kan*	Ammendola *et al*.,2007

Bacteria were grown at 37°C in Luria-Bertani (LB) liquid medium (1% bacto tryptone w/v, 0.5% yeast extract w/v, 1% NaCl w/v) or in LB medium solidified with 1.5% (w/v) agar. For growth under metal limiting conditions a modified M9 minimal medium, hereafter named modM9 (43 mM Na_2_HPO_4_, 22 mM KH_2_PO_4_, 19 mM NH_4_Cl, 1 mM MgSO_4_, 0.1 mM CaCl_2 _and 0.2% glucose) was used. To prepare the modM9, as well as other zinc-free solutions, we used ultra-pure water produced by a reverse osmosis system characterized by conductivity lower than 0.03 μS/cm. Moreover, bacterial culture and all solutions used with modM9 were prepared and incubated using zinc-free polypropylene plasticware (Falcon 50 and 10 ml tubes, Gilson tips and Eppendorf microtubes) avoiding glassware and other uncontrolled materials, except the 96-well plates used for the growth curves in modM9 which were in polystyrene. In this case, to remove metal contaminants of microtiter plates were treated overnight with 10 μM EDTA and then washed three times with fresh modM9 to eliminate EDTA traces. The effective ability of this procedure in removing zinc traces was evaluated by measuring the emission spectra of the final washing solution after the addition of 25 μM Zinquin, a highly specific Zn-fluorophore [17].

When required, the culture media were supplemented with the appropriate antibiotics (ampicillin 100 μg/ml, kanamycin 50 μg/ml, chloramphenicol 15 μg/ml).

### Mutant strains construction

All *E. coli *O157:H7 knockout mutants and the 3xFLAG strains were obtained following the protocol described by Datsenko and Wanner [28] and the epitope tagging method described by Uzzau *et al*. [29], respectively. The plasmids and the oligonucleotides used for mutants' construction are listed in Table 2 and 3, respectively. Recombinant strains were selected on chloramphenicol or kanamycin LB plates and confirmed by PCR using oligonucleotides internal to the chloramphenicol or kanamycin resistance cassettes in combination with primers specific for each gene.

**Table 2:** Plasmids

Plasmid	Relevant genotype or characteristic	Reference or source
pKD46	lambda red recombinase function	Datsenko and Wanner, 2000
pKD3	chloramphenicol resistance cassette template	Datsenko and Wanner, 2000
pKD4	kanamycin resistance cassette template	Datsenko and Wanner, 2000
pSUB11	3xFLAG-kanamycin resistance cassette template	Uzzau *et al*., 2001
p18ZnuAO157	ZnuA of E. *coli *O157:H7 cloned in pEMBL18	This work
p18ZnuA*E. coli*	ZnuA of E. *coli *K12 cloned in pEMBL18	This work

**Table 3:** Oligonucleotides used

Primer	Sequence(5'-3')
ZinT-HP1	TTTAGGTGTCTTTATTGTTAGCGCTCCTGCCTTTTCGCATTGTAGGCTGGAGCTGCTTCG
ZinT-HP2	CCACTTCTTCGCTACTCAACTGATATGGATAATACGTTGGCCCATATGAATATCCTCCTTAG
ZnuA-HP1	ATTATTCGCCGCTCTCTGGGGCGGTGCAACACAGGCCGCTGTAGGCTGGAGCTGCTTCG
ZnuA-HP2	CCTTTCAGGCAGCTCGCATACTGGTTGGCTAATTGACTCAGGCATATGAATATCCTCCTTAG
Zur-HP1	AAATCTGCGCGCAGCGTAATGTGCGCCTGACCCCACAGCTGTAGGCTGGAGCTGCTTCG
Zur-HP2	GCACAGAGTGATCATGCTGGCACTGTTCAGGATGACGACACGCCATATGAATATCCTCCTTAG
EtpC-HP1	ATAAAAGATATCGTACTTAAAATGCTGACGCCAAACCGGCTGTAGGCTGGAGCTGCTTCG
EtpC-HP2	CAGCAATAAATGCATCATATAACTGACCATCACGCTCGACCATATGAATATCCTCCTTAG
EtpD-HP1	TTTATGGTTTTGCTGTGGTCGATATGCACAACGGTATACTTGTAGGCTGGAGCTGCTTCG
EtpD-HP2	GCATAAAACGCAGCAATCGCCGCTTTCACCTTCCGGAAAGCATATGAATATCCTCCTTAG
ZinT-3xFLAGF	GTTGAGTAGCGAAGAAGTGGTCGAGGAAATGATGTCTCATGACTACAAAGACCATGACGG
ZinT-3xFLAGR	CTTTCTCTGTTGGCCGTATTGTGTATGGAATCCGTTATTGGCATATGAATATCCTCCTTAG
ZnuA-3xFLAGF	TCAATTAGCCAACCAGTATGCGAGCTGCCTGAAAGGAGATGACTACAAAGACCATGACGG
ZnuA-3xFLAGR	TGACAATTGGCGTGGCATCGCGGTGATAAACATAGGGCCGCATATGAATATCCTCCTTAG
ZnuA_O157_-Pst-F	AACTGCAGTGTCGACTTACCTGCG
ZnuA_O157_-Xba-R	GCTCTAGATTATTAAACGCCAGGGCGA
ZnuA _E *coli*_Kpn-F	GGGGTACCGTTACATAAAAAAACGCTTC
ZnuA _E *coli*_Xba-R	GCTCTAGATTAATCTCCTTTCAGGCAG
Clo-Int^a^	CTGGATATACCACCGTTGAT
Stop Clo-Int^a^	CACTCATCGCAGTACTGTT
Kan-F^a^	TGAACAAGATGGATTGCACG
Kan-R^a^	AAGAACTCGTCAAGAAGGC

P1, P2 and 3xFLAG homolog sequences are underlined

**a **primers used for screening of mutant clones or in Southern experiments

The double mutants (strains bearing double gene knockout or an epitope-flagged gene and a null mutation simultaneously) were constructed by a previously described procedure [27], electroporating the products of PCR reaction with primers specific for second mutation, in cells with the chromosome bearing the previous mutation. The resulting strains RG114, RG-F118, RG-F119, RG-F120, RG-F121, RG-F122, and RG-F123 were selected as mentioned above. To further verify the modification of the targeted genes, all mutant strains were checked also by Southern-blot procedure (data not shown).

Plasmids used for complementation assays were obtained by cloning the *znu*A gene from *E. coli *O157:H7 and *E. coli *K12 in pEMBL 18. The *znu*A sequences, including their promoter regions, were amplified by PCR using specific oligonucleotides (Table 3) and inserted in the XbaI and PstI (*E. coli *O157:H7) or XbaI and KpnI (*E. coli *K12) restriction sites of pEMBL 18. The resulting plasmids were called p18ZnuAO157 and p18ZnuA*E. coli *(Table 2).

### Growth curves

Each bacterial strain was grown overnight in LB broth at 37°C and then diluted 1:1000 in fresh LB, supplemented or not with 0.5 mM or 2 mM EDTA and 0.2, 0.5 and 1 mM ZnSO_4_. Aliquots of 300 μl of these dilutions were inoculated in 96-well plate (Becton-Dickinson) and incubated at 37°C with shaking. Growths in modM9 of each strain, including the RG113 bearing plasmid p18ZnuA O157 or p18ZnuA*E. coli*, were carried out by diluting preinocula 1:500 in fresh medium supplemented or not with 0.25, 0.5, 1 or 5 μM ZnSO_4_. Bacterial growth was monitored at 595 nm every hour for 15 hours using a microtiter-plate reader (Biotek instrument mod. ELX808). Assays were performed in triplicate and each strain was tested in three independent experiments.

### Complementation assay

Wild type, *znu*A deleted strain (RG113) and RG113 harbouring plasmids p18ZnuAO157 or p18ZnuAE. *coli *were grown overnight at 37°C in LB broth supplemented with the appropriate antibiotics, diluted to 1 OD_600 _and then streaked on LB plates containing 0, 0.5, 1 and 2 mM EDTA with or without antibiotics. Bacterial ability to form visible colonies on these plates was analyzed after 24 hours of incubation at 37°C.

### Western blot analysis

The expression of *zin*T and *znu*A was indirectly analyzed by measuring the intracellular accumulation of the epitope-tagged proteins. Strains carrying the epitope-tagged genes were grown at 37°C in LB or in modM9 in presence or absence of EDTA or transition metals. Bacteria cultivated in LB were exposed to 0.5 mM EDTA and 0.2 mM ZnSO_4_, or 0.25 mM CdSO_4_, whereas bacteria in modM9 were grown in presence or not of 5 μM EDTA and of 5 μM ZnSO_4_, FeSO_4_, CuSO_4 _or MnCl_2_. After 4 h of growth in LB and 6 h or 16 h in modM9, aliquots of 2×10^8 ^cells were harvested by centrifugation, lysed in sample buffer containing sodium dodecyl sulphate (SDS) and β-mercaptoethanol and boiled for 8 min at 100°C.

Extracellular ZinT was prepared by filtering through a 22 μm-pore size filter (Millex, Millipore) the supernatant from a volume of culture containing 5×10^8 ^cells. Extracellular proteins were concentrated to 100 μl by Amicon ultra centrifugal filter devices (10,000 NMWL-Millipore) and incubated overnight at -20°C in 1 ml ice-cold acetone. Each pellet, obtained after 10 min centrifugation at 13,000 × g at 4°C, was resuspended in 10 μl of Lysis Buffer (1 mM EDTA, 100 mM NaCl, 50 mM Tris-HCl, pH 8.0).

Proteins were separated by 12% SDS-PAGE and blotted onto nitrocellulose membranes (Hybond C, Amersham). The epitope-flagged proteins were revealed by anti-FLAG M2 monoclonal antibody (Sigma-Aldrich) as primary antibody and anti-mouse HRP-conjugated IgG (Bio-Rad) as secondary antibody. Native ZinT was revealed by rabbit anti ZinT polyclonal antibody (produced by AnaSpec using the synthetic peptide CDYDGYKILTYKSGK) as primary antibody, and goat anti-rabbit HRP-conjugated IgG (Bio-Rad) as secondary antibody. Detection was performed by enhanced chemiluminescence (ECL Advance, Amersham).

### Studies on ZinT import and preparation of apo and zinc containing-ZinT

A deleted *zin*T strain (RG-F120) was grown overnight in LB and diluted 1:500 in fresh broth and incubated at 37°C until to OD_600 _= 0.5. Subsequently, 25 or 0.25 μg of extracellular tagged-ZinT, derived from the supernatant culture of RG-F116 strain (grown in modM9 for 6 h as described in Western-blot analysis), were mixed to 5×10^8 ^cells and incubated in LB or LB supplemented with 0.5 mM EDTA at 37°C without shaking. At starting point or after 4 h of incubation the cells were washed three times in PBS to remove external ZinT. Total extracts were analyzed by Western blot.

In order to prepare the apo or the holo form of ZinT, extracellular ZinT was isolated from the culture supernatants of the RG-F116 strain grown in modM9 for 6 h at 37°C. Zinc was removed from ZinT by dialysis against 2 mM EDTA, 50 mM acetate buffer, pH 5.4, for 24 h. Subsequently, the protein was dialyzed for 24 h against 100 mM NaCl, 50 mM acetate buffer, pH 5.4 to remove excess EDTA and finally against 50 mM Tris-HCl, pH 6.0. The solutions used for the dialyses of apo-ZinT were prepared with ultra-pure water (0.03 μS/cm) in nitric acid-treated glassware.

To prepare holo-ZinT, the apo-ZinT protein was dialyzed for 24 h against 1 mM ZnSO_4_, 50 mM Tris-HCl, pH 7.5, and then extensively dialyzed against 50 mM Tris-HCl, pH 7.5. Protein concentration was evaluated by the method of Lowry [30].

### Cell cultures and competition assay

Human epithelial colorectal adenocarcinoma cells (Caco-2) were cultured at 37°C in humidified air with CO_2_. Caco-2 cell line was maintained in Dulbecco's modified Eagle's medium (D-MEM) containing 1 g/l glucose, 100 μg/ml penicillin, 100 μg/ml streptomycin, 4 mM L-glutamine and 10% fetal calf serum.

For adhesion experiments *E. coli *O157:H7 wild type and mutant strains were grown in LB broth supplemented with 2 mM EDTA. Overnight cultures were diluted in D-MEM to a final concentration of 10^6 ^cells/ml and then 1 ml of this dilution was used to infect Caco-2 cells previously seeded on a 24-well plate. After two hours of infection each well was washed three times with phosphate buffered saline (PBS), to remove non adherent bacteria, and then lysed with cold Triton X-100 solution (0.5% in PBS). Serial dilutions of the cellular lysates were plated on LB containing kanamycin or chloramphenicol (see Table 4) to enumerate adherent bacteria. The same approach was used to carry out competitive infections. In this case, the 10^6 ^cells/ml bacterial suspensions in D-MEM were mixed in pairs in a 1:1 ratio and 1 ml of these mixtures was used to infect Caco-2 cells. Each competition experiment was performed in five different wells and repeated tree times. The infected cells were treated as described above and, after plating of the adherent bacteria, 200 colonies were individually picked on selective plates. The competitive index (CI) was calculated by the formula CI = output (Strain A/Strain B)/inoculum (Strain A/Strain B). Statistical differences between outputs and inputs were determined by the Student's *t*-test.

**Table 4:** Competition assays in CaCo-2 cells

Strain A (relevant genotype)	Strain B (relevant genotype)	Median CI^a^	P^b^
Wild type	znuA::cam*	6.833	0.034
Wild type	zinT::kan*	0.980	NS
Wild type	zinT:: kan znuA:: cam*	3.899	0.004
*zinT::kan*	zinT:: kan znuA:: cam*	2.788	< 0.001
*znuA::cam*	zinT:: kan* znuA:: cam	0.697	0.004

**a**. Competitive index = output (Strain A/Strain B)/inoculum (Strain A/Strain B).

**b**. Statistical differences between output and inocula (the P-values) were determined by the Students *t *test. NS, not significant.

* Antibiotic used for strains selection

To analyse the expression of ZnuA and ZinT during infections, Caco-2 cells infected with the RG-F116 or the RG-F117 strains (which express epitope-tagged ZnuA and ZinT, respectively) were lysed 2 h post-infection, and the lysates were harvested and analysed by Western blot.

## Results

### Influence of *zin*T and *znu*A on *E. coli *O157:H7 growth

**Figure 1:**
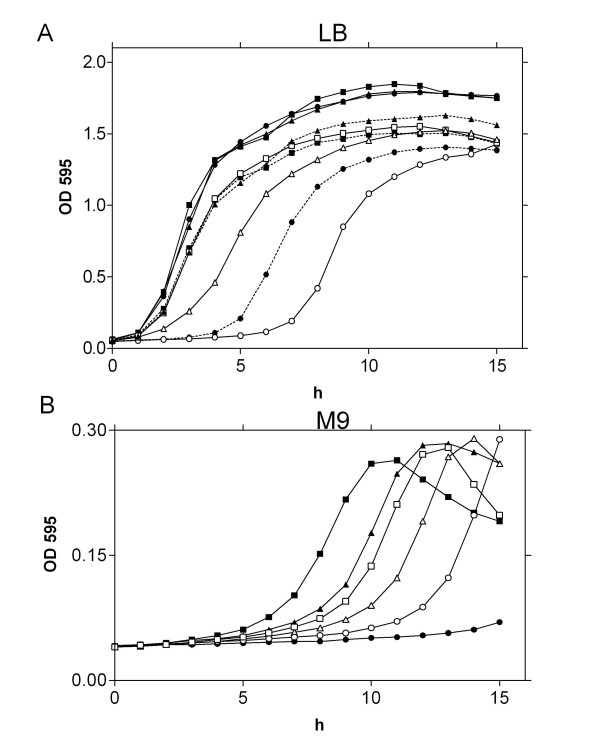
**Growth curves**. Panel A: growth curves of wild type (squares), Δ*zin*T::*kan *(triangles) and Δ*znu*A::*kan *(circles) in LB medium (close symbols), in LB supplemented with 0.5 mM EDTA (open symbols) and 0.2 mM ZnSO_4 _(dotted lines). Panel B: growth curves of the same strains in modM9 (close symbols) and in modM9 supplemented with 5 μM ZnSO_4 _(open symbols).

We compared the growth of the wild type strain to that of mutant strains deleted of *zin*T (RG112), *znu*A (RG113) or both the genes (RG114). No differences in growth were observed when bacteria were cultivated in LB, whereas the growth of all mutant strains decreased with 0.5 mM EDTA (Figure 1, panel A, data not shown for RG114) and even more with 2 mM EDTA treatment (data not shown). A recovery in growth of all mutant strains was observed upon supplementation of ZnSO_4 _to the LB containing EDTA.

In modM9 all mutant strains displayed a clear growth defect with respect to the wild type strain (Figure 1, panel B), with a major impairment of the growth of strains lacking *znu*A (RG114 data not shown) than that of the strain lacking only *zin*T. In this case, however, the addition of ZnSO_4 _to the culture medium significantly reduced the rate of growth of the wild type (Additional file 1: Figure S1, panel A) and *zin*T mutant strains, likely due to toxic effects of the extracellular metal. In contrast, a clear improvement in the growth of the strains lacking *znu*A was observed upon the addition of zinc to the medium (Figure 1, panel B and Additional file 1: Figure S1, panel B). The growth defect of the *znu*A mutant strain was complemented by a multicopy plasmid overexpressing *E. coli *ZnuA, indicating that disruption of *znu*A does not abolish the functionality of the other genes of the *znu*ABC operon (Table 5 and Additional file 2: Figure S2). The reduced rate of growth of the complemented strains is likely due to gene dosage effects, as previously described [17].

**Table 5:** Growth on LB plates

Strains^a^	EDTA concentration			
	0	0.5 mM	1 mM	2 mM
WT	++	++	++	++
RG113 (Δ*znuA*:: *kan*)	++	+/-	+/-	-
RG113 + p18ZnuAO157	++	+	+	+
RG113 + p18ZnuA*E. coli*	++	+	+	+

**a **The strains were grown overnight in LB medium and then streaked on LB plates containing the indicated amounts of EDTA. Growth on agar plates was not modified by the presence or absence of antibiotics.

**Symbols**: ++ growth, + weak growth, +/- weak growth of very small colonies, - no growth.

### ZinT and ZnuA expression studies

**Figure 2:**
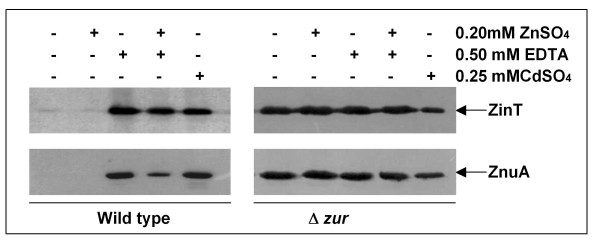
**ZinT and ZnuA accumulation in *zur *wild type and in *zur *deleted strains**. RG-F116 (*zin*T::3xFLAG-*kan*), RG-F117 (*znu*A::3xFLAG-*kan*), RG-F118 (Δ*zur*::*cat zin*T::3xFLAG-*kan*) and RG-F119 (Δ*zur*::*cat znu*A::3xFLAG-*kan*) strains were grown for 4 h in LB medium in presence or absence of 0.2 mM ZnSO_4_, 0.5 mM EDTA or 0.2 mM CdSO_4 _as indicated. The extracts were analyzed by Western blot.

The expression of *zin*T and *znu*A was indirectly analyzed by monitoring the proteins accumulation in strains which were modified by introducing the sequence encoding the 3xFLAG epitope at the 3'end of each gene (Figure 2). In agreement with previous studies [18,21], also in *E. coli *O157:H7 cadmium and EDTA were able to induce the expression of ZinT and ZnuA. Moreover, ZnuA accumulation drastically decreased when bacteria were grown in 0.5 mM EDTA in presence of 0.2 mM ZnSO_4_, a quantity unable to saturate the binding ability of the chelator, whereas ZinT accumulation was only moderately affected. Higher zinc concentrations, however, were able to completely abolish ZinT and ZnuA accumulation (data not shown). In addition, the tagged proteins accumulated both in standard LB and in LB supplemented with zinc in *zur *deleted strains, confirming that *zin*T and *znu*A are negatively regulated by Zur, as already observed in other bacteria in previous studies [4,12,18,31,32].

**Figure 3:**
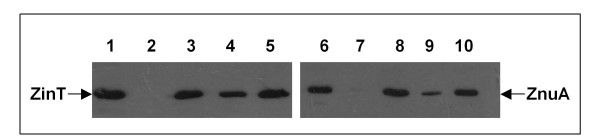
**Influence of metals on ZinT and ZnuA accumulation**. RG-F116 (*zin*T::3xFLAG-*kan*) and RG-F117 (*znu*A::3xFLAG-*kan*) strains were grown for 16 h in modM9 (lanes 1 and 6) in presence of ZnSO_4 _(lanes 2 and 7), FeSO_4 _(lanes 3 and 8), CuSO_4 _(lanes 4 and 9) or MnCl_2 _(lanes 5 and 10). Metal concentration was 5 μM. The extracts were analyzed by Western blot.

**Figure 4:**
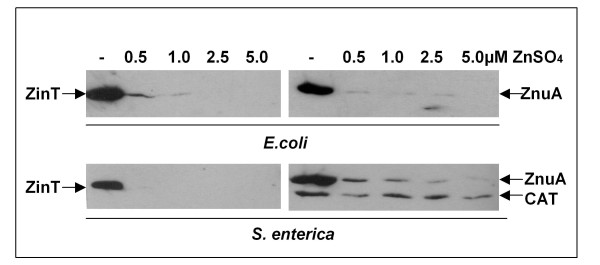
**Zinc-dependent ZinT and ZnuA accumulation in *E. coli *O157:H7 and *S. enterica *strains**. RG-F116 (*zin*T::3xFLAG-*kan*), RG-F117 (*znu*A::3xFLAG-*kan*) *E. coli *O157:H7 strains or PP134 (*zin*T::3xFLAG-*kan*) and SA140 (*znu*A::3xFLAG- *kan ilv*I::Tn10dTac-*ca*t:: 3xFLAG- *kan*) *S. enterica *strains were grown for 16 h in modM9 supplemented or not with various concentrations of ZnSO_4_, as indicated. The extracts were analyzed by Western blot. In SA140 strain the chloramphenicol acetyltransferase (CAT) was used as an internal standard.

To evaluate the specificity of the response of *zin*T and *znu*A to metal ions, the accumulation of the two proteins was analyzed in modM9 supplemented with 5 μM ZnSO_4_, FeSO_4_, CuSO_4 _or MnCl_2_. The expression of both genes was repressed by zinc (Figure 3) whereas, in contrast to the results obtained with *S. enterica *[17], *znu*A and, to a lesser extent, *zin*T expression was partially inhibited by copper. Small differences in the regulation of the Zur-regulated genes between *E. coli *O157:H7 and *S. enterica *(PP134 and SA140) were also suggested by a titration of protein accumulation in response to external zinc (Figure 4). In *E. coli *O157:H7 strains the two genes were similarly expressed, with a slightly higher ZinT accumulation in presence of 0.5 μM ZnSO_4_. In contrast, in *S. enterica *only ZnuA was detectable at this zinc concentration.

**Figure 5:**
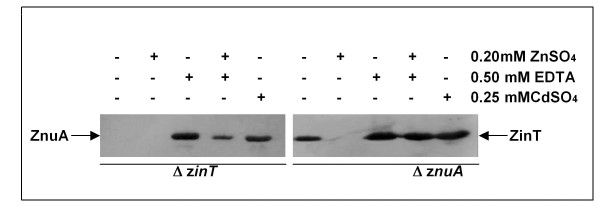
**Different accumulation of ZinT and ZnuA in the deleted strains in LB medium**. RG-F120 (Δ*zin*T::*cat znu*A::3xFLAG-*kan*) and RG-F121 (Δ*znu*A::*cat zin*T::3xFLAG-*kan*) strains were grown for 4 h in LB medium in presence or absence of 0.2 mM ZnSO_4_, 0.5 mM EDTA or 0.25 mM CdSO_4_, as indicated. The extracts were analyzed by Western blot.

**Figure 6:**
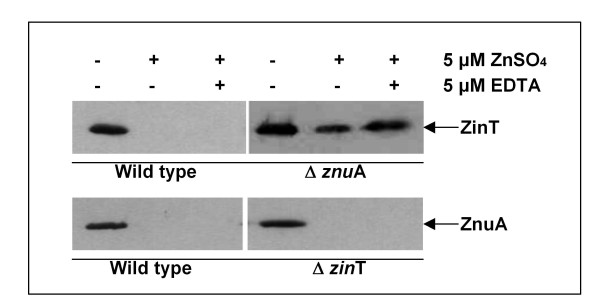
**Different accumulation of ZinT and ZnuA in the deleted strains in modM9 medium**. The wild type strains RG-F116 (*zin*T::3xFLAG-*kan*) and RG-F117 (*znu*A::3xFLAG-*kan*), and the deleted strains RG-F120 (Δ*zin*T::*cat znu*A::3xFLAG-*kan*) and RG-F121(Δ*znu*A::*cat zin*T::3xFLAG-*kan*) were grown for 16 h in modM9 in presence or absence of 5 μM ZnSO_4 _or 5 μM EDTA, as indicated. The extracts were analyzed by Western blot.

The accumulation of the tagged-proteins was analyzed also in mutant strains deleted of *zin*T (RG-F120) or of *znu*A (RG-F121). Figure 5 shows that ZnuA accumulation in the strain lacking a functional *zin*T was comparable to that observed in the wild type strain in the same conditions (see Figure 2). In contrast, ZinT was expressed by the RG-F121 strain either in LB, where it was normally absent (Figure 5), or in modM9 supplemented with zinc (Figure 6). These observations support the hypothesis that the role of ZinT in zinc homeostasis is dependent on the presence of ZnuA and that this protein is not able to directly deliver zinc to ZnuB.

### Extracellular ZinT

**Figure 7:**
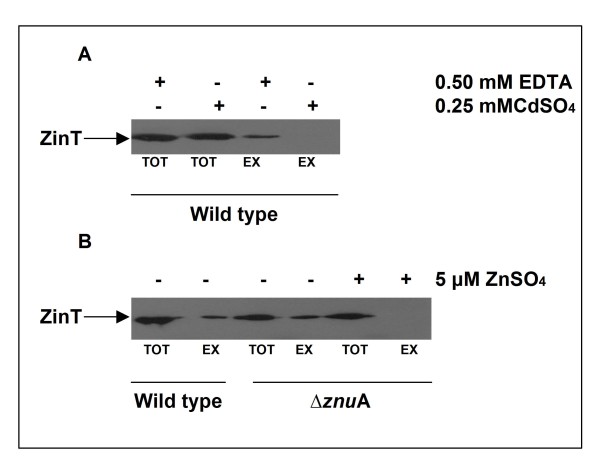
**Extracellular ZinT accumulation**. Panel A: RG-F116 (*zin*T::3xFLAG-*kan*) strain was grown in LB medium supplemented with 0.5 mM EDTA (lanes 1 and 3) or with 0.25 mM CdSO_4 _(lanes 2 and 4). After 4 h of growth, total (lanes 1 and 2) or extracellular extracts (lanes 3 and 4) were loaded on SDS-PAGE and analyzed by Western blot. Panel B: RG-F116 (lanes 1 and 2) and RG-F121 (Δ*znu*A::*cat zin*T::3xFLAG-*kan*) strains (lanes 3, 4, 5 and 6) were grown in modM9 (lanes 1, 2, 3 and 4) or supplemented with 5 μM of ZnSO_4 _(lanes 5 and 6). After 6 h of growth, total (lanes 1, 3 and 5) or extracellular extracts (lanes 2, 4 and 6) were loaded on SDS-PAGE and analyzed by Western blot.

In a previous work ZinT was identified in the culture supernatant of *E. coli *O157:H7 strain and suggested to be a substrate of the type 2 secretion system (T2SS) [23], whereas no studies have yet examined the possibility that ZnuA could be secreted. To investigate this possibility and better characterize ZinT export, total or extracellular extracts from RG-F116 and RG-F117 strains were analyzed. Strains were grown in LB supplemented with 0.5 mM EDTA or 0.25 mM CdSO_4 _for only 4 h to prevent the possible release of proteins in the culture medium by lysis of starved bacterial cells. In none of the tested conditions could ZnuA be detected in the culture supernatant (data not shown). In contrast, as shown in Figure 7 panel A ZinT was detectable in the extracellular fraction of bacteria grown in presence of EDTA but not in that of bacteria cultivated in presence of cadmium, suggesting that the secretion was not possible for Cd-containing ZinT while the sequestration of metals by EDTA likely produced an apo-form able to be secreted outside the cell.

To verify if protein secretion was prevented by metal binding, ZinT was produced in the RG-F121 strain grown in modM9, supplemented or not with 5 μM ZnSO_4 _(Figure 7, panel B). This strain was chosen because the absence of *znu*A allows the expression of *zin*T in modM9 also in presence of zinc, an essential condition to carry out the proposed experiment. As expected, an expression band was not visible in the supernatant obtained in presence of zinc whereas this band was observable in absence of the metal for RG-F116 and RG-F121 strains. Additional file 3: Figure S3 shows that *E. coli *O157:H7 secretes only a very limited number of proteins in modM9 and that there is not an evident release of intracellular proteins.

**Figure 8:**
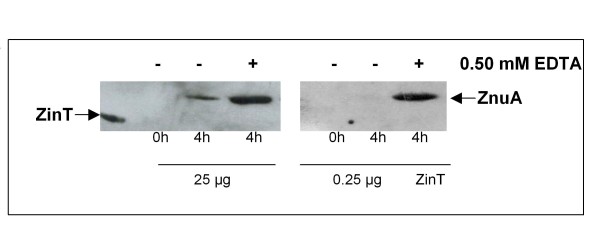
**Influence of extracellular ZinT on z*nu*A expression**. RG-F120 (Δ*zin*T::*cat znu*A::3xFLAG-*kan*) strain was grown in LB medium (lanes 2, 3, 5 and 6) or LB supplemented with 0.5 mM EDTA (lanes 4 and 7) in presence of 25 μg (lanes 2, 3 and 4) or 0.25 μg (lanes 5, 6 and 7) extracellular ZinT. The extracts, analyzed by Western blot, were prepared after a 4 h growth (lanes 3, 4, 6 and 7), or immediately after the addition of extracellular ZinT (lanes 2 and 5), as negative control. 25 μg of extracellular ZinT was loaded in lane 1 as positive control.

In an attempt to identify a role for extracellular ZinT, we investigated the possibility that secreted ZinT could rebind to the bacterial cell. Cultures of RG-F120 strain, bearing a gene encoding a tagged-ZnuA and a deletion in *zin*T, were incubated for 4 h with extracellular tagged-ZinT obtained from the supernatant culture of RG-F116 strain grown in modM9 for 6 h. Subsequently, cellular extracts were analyzed by Western blot to examine the fate of ZinT, using tagged ZnuA as positive control. As shown in Figure 8, when RG-F120 was grown in LB or in LB with 0.5 mM EDTA in presence of 25 μg of extracellular ZinT the protein was not found in association with the bacterial cell. Unexpectedly, we observed that extracellular ZinT induced the accumulation of ZnuA in LB (Figure 8 lane 3), where this protein was hardly detectable (see Figure 2). Such induction of *znu*A gene was not observed (Figure 8 lane 6) in bacteria incubated in presence of a hundredfold lower amount of extracellular ZinT (0.25 μg), suggesting that ZnuA accumulation could be due to the ability of extracellular ZinT to sequester external zinc. To verify this possibility, the experiment was repeated using either apo- or zinc-containing ZinT. ZnuA accumulation appeared in LB only when the apo-form (data not shown) was used, showing the similar expression pattern obtained with the extracellular ZinT produced in modM9. These results indicated that apo-ZinT sequesters environmental zinc thus inducing the *zur *regulon, and that extracellular ZinT released by bacteria grown in modM9 is mainly in the zinc-free form, as already indicated by results described in Figure 7.

In order to obtain strains unable to secrete ZinT we used the RG-F116 strain to delete *etp*C (RG-F122) or *etp*D (RG-F123), the first two genes of the operon of T2SS [33]. Contrary to our expectations, tagged-ZinT was detected in the supernatant of these mutants grown in LB supplemented with 0.5 mM EDTA and its accumulation was comparable to that observed in the wild type strain (data not shown). To exclude that the FLAG-epitope tail could interfere with the export of the protein, we have grown the *etp*C null mutant strain (RG115), where the *zin*T gene was unmodified, under the same experimental conditions. The observation of a band in extracellular extracts, revealed by anti ZinT polyclonal antibody as primary antibody, suggested that T2SS was not the main secretion system for the export of the protein encoded by chromosomal *zin*T (data not shown). Extracellular ZinT was also revealed in the culture supernatant of *E. coli *K12 (DH5α) and B (BL21) strains, by using the same anti ZinT polyclonal antibody (data not shown). This result supports the hypothesis that ZinT is not secreted by T2SS, as in the laboratory strains of *E. coli *the T2SS is transcriptionally silenced by the histone-like nucleoid-structuring protein H-NS [34,35].

### Effects of *zin*T and *znu*A deletion on *E. coli *O157:H7 adhesion to Caco-2 cells

**Figure 9:**
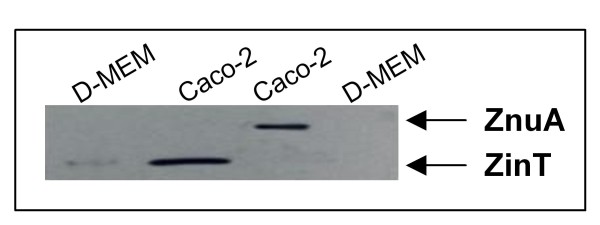
**ZinT and ZnuA accumulation in *E. coli *O157:H7 adherent to epithelial cells**. ZinT and ZnuA accumulation of RG-F116 (*zin*T::3xFLAG-*kan*) and RG-F117 (*znu*A::3xFLAG-*kan*) strains, grown overnight in D-MEM (lanes 1 and 4), was compared to accumulation of proteins in bacteria recovered from infected Caco-2 cells (lanes 2 and 3).

It has previously been reported that inactivation of *zin*T has a dramatic effect on the ability of *E. coli *O157:H7 to adhere to HeLa cells [23]. To investigate the relevance of the zinc import apparatus in the *E. coli *O157:H7 interaction with host cells, we have initially analyzed ZnuA and ZinT accumulation in bacteria (RG-F116 and RG-F117) adhering to Caco-2 epithelial cells. Results reported in Figure 9 indicate that in presence of Caco-2 cells both proteins were expressed at levels that were significantly higher than those observed in bacteria grown in D-MEM. This observation suggests that Caco-2 cells deplete the medium of zinc or that the cell surface microenvironment is poor of zinc. Despite this finding and unlike the results obtained by Ho *et al*. [23] with HeLa cells under slightly different experimental conditions, we were unable to demonstrate that inactivation of *znu*A or *zin*T significantly decreases the ability of *E. coli *O157:H7 to adhere to Caco-2 epithelial cells with respect to the wild type strain (data not show). However, as the number of adherent bacteria was highly variable in different experiments, to better appreciate the contribution of ZnuA and ZinT to the interaction of *E. coli *O157:H7 with Caco-2 cells, we carried out adhesion experiments using mixtures of different strains (Table 4). These competition experiments revealed that mutant strains lacking *znu*A (RG113 and RG114) were significantly disadvantaged compared to the wild type strain but failed to identify an adherence defect in the strain lacking only *zin*T (RG112). It is worth nothing that the loss of adherence ability of the *znu*A mutant strain is not trivially due to a reduced ability to grow in D-MEM. In fact, co-cultures of the wild type and of the *znu*A mutant revealed that the two strain grow equally well in this medium, indicating that it is likely rich in zinc (data not shown). Moreover, the results reported in Additional file 4: Table S1 show that during co-infection experiments the *znu*A mutant strain replicated more efficiently than the wild type strain, despite it was less able to adhere to the epithelial monolayer. In addition, we observed that the *zin*T/*znu*A mutant strain (RG114) was more able to adhere to epithelial cells than the single *znu*A mutant. This result, which replicates a comparable finding in Salmonella [17], could be tentatively explained by a toxic effect of ZinT in the absence of ZnuA, due to its ability to sequester zinc without being able to transfer the metal to the ZnuB permease.

## Discussion

The results reported in this work confirm the central importance of the ZnuABC transporter in the process of zinc uptake also in *E. coli *O157:H7. In fact, growth of strains lacking *znu*A, the gene encoding for the periplasmic component of the transporter, is severely impaired in media poor of zinc (LB supplemented with EDTA or modM9), but is identical to that of the wild type strain in LB medium where zinc is abundantly available (Figure 1). The growth impairment of *znu*A mutant strains is clearly attributable to the lacking of this gene because it is complemented by plasmids harbouring the *znu*A copy (Table 5 and Additional file 2: Figure S2). In line with these observations, ZnuA accumulates in bacteria grown in zinc-limiting conditions but is hardly detectable in bacteria recovered from LB (Figures 2 and 5). Accumulation of ZnuA is regulated by zinc and not by manganese or iron as shown in Figure 3. However, in line with previous observation by the group of Kershaw [36] on *E. coli *K12 and in contrast to results obtained on *S. enterica *[17], it is somehow modulated by copper. We believe that it is unlikely that ZnuABC participates to the mechanisms of copper homeostasis and we suggest that this effect could be explained by the very similar properties of the copper and zinc atoms which likely allow the accommodation of copper in the zinc binding site of Zur.

The results reported in this work provide further evidences that also ZinT participates in the mechanisms of zinc uptake, in line with recent studies [18,24,25]. We have verified that also in *E. coli *O157:H7 *zin*T is regulated by Zur and that it is induced under conditions of zinc deficiency. The absence of *zin*T has no discernable effects on bacterial replication in rich media, but significantly affects growth either in presence of chelating agents or in modM9 (Figure 1). However, unlike what observed for the *znu*A mutant, zinc supply does not clearly improve the growth of the *zin*T mutant in modM9 and we could not observe an additive effect of the double mutation *zin*T*/znu*A. These observations corroborate the suggestion that the role of ZinT in zinc uptake is subordinated to that of ZnuA and that zinc ions bound by ZinT are subsequently transferred to ZnuA, which ensures zinc import in the cytoplasm [18]. This consideration is in agreement with the observation that *zin*T is constitutively expressed in a *znu*A mutant strain, but that ZnuA accumulation is not significantly modulated by the absence of *zin*T (Figure 5). This is likely explained by a decrease of the zinc concentration in the cytoplasm in the absence of ZnuA, but not of ZinT, with the consequent derepression of *zin*T by Zur.

It should be highlighted that the *zin*T mutant strain exhibits a sharp growth defect either in LB supplemented with 0.5 mM EDTA or in defined medium. This behaviour was not observed in a *zin*T mutant of *S. enterica *[18], which showed a clear impairment of growth in LB only in presence of 2 mM EDTA, a concentration at which the *E. coli *O157:H7 mutant is hardly able to grow. Furthermore, our results indicate that there are differences between *E. coli *O157:H7 and *S. enterica *in the regulation of *znu*A and *zin*T in response to low zinc availability (Figure 4). In particular, in *E. coli *O157:H7 ZinT can be easily detected in bacteria growing in a medium supplemented with up to 1 μM zinc, whereas in *S. enterica *this protein accumulates only in media completely devoid of the metal. This observation, which is in agreement with the different effect of *zin*T disruption in the two bacterial species, may suggest that the relative role of ZnuA and ZinT could be slightly different in the two microorganisms.

Although several of the bacteria which rely on the ZnuABC transporter to import zinc do not possess ZinT [18], our study suggests that, despite the role of ZinT is clearly dependent on the presence of ZnuA, its contribution to metal recruitment within the periplasmic space is considerable. The exact involvement of ZinT in zinc uptake is yet to be determined, but it is possible to hypothesize that ZinT and ZnuA display a diverse ability to sequester metal ions from different molecules within the periplasm or that the binding of ZinT to ZnuA accelerates the rate of metal transfer to ZnuB [18].

We have also analyzed the involvement of the zinc uptake system in the interaction between *E. coli *O157:H7 and epithelial Caco-2 cells. Both ZnuA and ZinT accumulates at high levels in bacteria adhering to the cell monolayer, but not in bacteria cultivated in D-MEM without cells (Figure 9). This finding expands previous observations showing that bacterial pathogens have to face with a problem of zinc paucity within the host [17] and specifically suggests that the host cell surface microenvironment is poor of zinc, possibly due to active metal sequestration mechanism implemented by eukaryotic cells. In line with this observation strains lacking *znu*A display a reduced ability to adhere to epithelial cells (Table 4). We could not observe significant alterations in the adhesion ability of the *zin*T mutant strain nor an additive effect of the *zin*T*/znu*A mutations, confirming the subordinate role of ZinT already revealed by the analysis of growth curves *in vitro *(Figure 1). This last finding is in contrast with the recent results reported by Ho and colleagues [23] who analyzed the role of YodA (ZinT) in the *E. coli *O157:H7 strain EDL933, observing that the *zin*T mutant strain exhibits a dramatic reduction in its ability to adhere to HeLa cells and to colonize the infant rabbit intestine [23]. Furthermore, they observed a reduction in growth of the *zin*T mutant also in LB medium. In principle, divergences between these two studies could due to genotypic differences between the strains employed or to differences in the *E. coli *ability to interact with different eukaryotic cell lines. However, it is worth nothing that the reduction in growth of the *zinT *mutant in LB medium observed by Ho *et al*. is unexpected on the basis of the presumed role of ZinT in zinc import and that, in line with the here reported results, *zin*T mutants of *S. enterica *[18] and *E. coli *K12 [24,25] grow as well as the wild type parental strains in zinc replete media. Moreover, Ho and colleagues identified ZinT even in the culture supernatants of *E. coli *O157:H7 strain and suggested that it is a substrate of the type 2 secretion system (T2SS) [23]. We have confirmed that a fraction of ZinT is actually exported selectively (ZnuA is not secreted) in the culture medium (Figure 7), but we failed to validate the suggestion that the secretion of this protein is facilitated by T2SS. In fact, ZinT is exported with comparable efficiency by the wild type strain or by mutant strains lacking *etp*C or *etp*D genes which encode for two different components of the T2SS gene cluster [33]. Moreover, we observed that ZinT is secreted also in *E. coli *K12 and B strains. This observation strongly argues against the involvement of T2SS in the export of ZinT because the genes encoding for the T2SS system are not expressed in *E. coli *K12 due to the repression by the histone-like nucleoid-structuring protein H-NS [34,35]. We hypothesize that the different result obtained by Ho et *al*. could be explained by their choice to analyze the secretion of ZinT in a strain overexpressing a V5-tagged ZinT. The T2SS might be involved in the recognition of this specific tag or in the secretion of proteins when overexpressed [37]. In any case, the T2SS system seems not to participate in the secretion of chromosomally encoded ZinT.

We have demonstrated that ZinT can be exported in the extracellular environment only in the metal free form. In fact, when ZinT is constitutively expressed in bacteria grown in media containing cadmium or zinc, it can not be identified in the culture supernatants, despite it is present in the periplasmic space (Figure 7). The release of metal-free ZinT in the extracellular environment may influence properties of the bacterial or host cells. This possibility is partially supported by the experiment showing that apo-ZinT, unlike the zinc containing protein, is able to influence *znu*A expression when provided externally to bacterial cells (Figure 8). The observed accumulation of ZnuA is likely due to the ability of ZinT to sequester the free zinc present in the culture medium, inducing a condition of zinc starvation. Although we have analyzed the effects of extracellular ZinT only on the bacterial cell, we hypothesize that the sequestration of extracellular zinc may have effects also on the host cells. In this view, it is interesting to note that several bacteria produce metal binding proteins located on the cell surface which mediates the microbial attachment to the human extracellular matrix. Proteins of this class include, for example, the laminin binding proteins (LBP) from *Streptococcus agalactiae *or *Streptococcus pyogenes*, which are structurally related to ZnuA [38,39]. Although the details of the interaction of LBP with laminin are still to be clarified, it is likely that LBP acts as an adhesin which binds to the zinc containing laminin in a metal-mediated manner. By analogy, we suggest that extracellular ZinT may interact with zinc-containing proteins in the intestinal epithelia, thus favouring *E. coli *O157:H7 colonization, or that its capability to sequester zinc ions from the environment may damage epithelial cells ability to neutralize bacterial adhesion.

## Conclusions

This study demonstrates that the high affinity ZnuABC uptake system plays a key role in zinc uptake in *E. coli *O157:H7 and that ZinT is an additional component of this metal transport system which significantly enhances the rate of metal uptake. In addition, our data indicate that the functionality of this transporter may influence the adhesion of bacteria to epithelial cells. These findings improve our knowledge about the importance of zinc in bacterial physiology and its role in the host-microbe interaction.

## Authors' contributions

RG and RS coordinated the study, participated to the manuscript preparation, carried out *E. coli *O157:H7 mutants construction, performed growth curves, complementation assay and *in vitro *expression studies, PP carried out studies with cultured cells, SA collaborated in the preparation of strains and to the set up of zinc free media, AB and LN participated in the design of the study and in the writing of the manuscript. All authors read and approved the final manuscript.

## Supplementary Material

Additional file 1 **Figure S1: Influence of zinc on modM9 growth curve**. The figure shows the growth curves of wild type and D*znu*A::*kan *strains in modM9 supplemented with various concentrations of ZnSO_4 _(0.25 mM, 0.5 mM, 1 mM and 5 mM). Click here for file

Additional file 2 **Figure S2: Growth curve of the complemented D*znu*A::*kan *strain in modM9**. The figure shows as the growth curves of D*znu*A::*kan *containing the plasmid p18ZnuAO157 or p18ZnuAE. *coli *are improved respect to that of D*znu*A::*kan*. Click here for file

Additional file 3 **Figure S3: Expression pattern of *zin*T in SDS-PAGE**. The figure shows the total extracellular extracts of *zin*T::3xFLAG-*kan *analysed by SDS-PAGE and stained by Coomassie- Blue or revealed by Western blot. Click here for file

Additional file 4 **Table S1: Competition assays in CaCo-2 cells**. The table shows as during co-infection experiments the *znu*A mutant strain replicated more efficiently than the wild type strain. Click here for file
