# Supplementary material for: Role of ZnuABC and ZinT in Escherichia coli O157:H7 zinc acquisition and interaction with epithelial cells
Source: BMC Microbiol. 2011 Feb 21;11:36. doi: 10.1186/1471-2180-11-36 (PMC3053223; doi:10.1186/1471-2180-11-36)
Supplement: Additional file 1 — Figure S1: Influence of zinc on modM9 growth curve. The figure shows the growth curves of wild type and DznuA::kan strains in modM9 supplemented with various concentrations of ZnSO4 (0.25 mM, 0.5 mM, 1 mM and 5 mM). [file 1471-2180-11-36-S1.PPTX]

## Slide 1
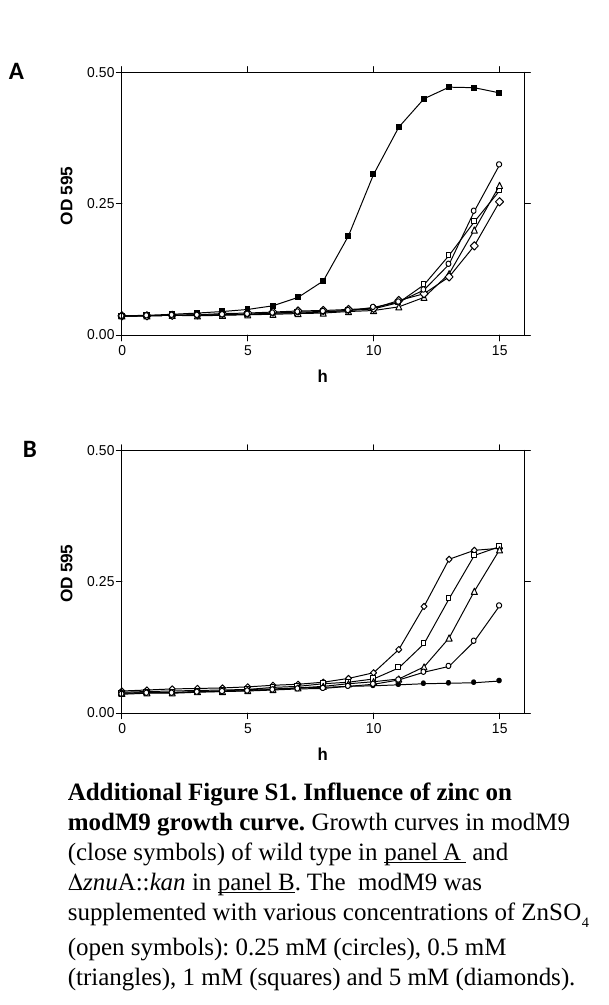

A
B
Additional Figure S1. Influence of zinc on modM9 growth curve. Growth curves in modM9 (close symbols) of wild type in panel A and ΔznuA::kan in panel B. The modM9 was supplemented with various concentrations of ZnSO4 (open symbols): 0.25 mM (circles), 0.5 mM (triangles), 1 mM (squares) and 5 mM (diamonds).
