# Supplementary material for: Role of ZnuABC and ZinT in Escherichia coli O157:H7 zinc acquisition and interaction with epithelial cells
Source: BMC Microbiol. 2011 Feb 21;11:36. doi: 10.1186/1471-2180-11-36 (PMC3053223; doi:10.1186/1471-2180-11-36)
Supplement: Additional file 2 — Figure S2: Growth curve of the complemented DznuA::kan strain in modM9. The figure shows as the growth curves of DznuA::kan containing the plasmid p18ZnuAO157 or p18ZnuAE. coli are improved respect to that of DznuA::kan. [file 1471-2180-11-36-S2.PPT]

## Slide 1
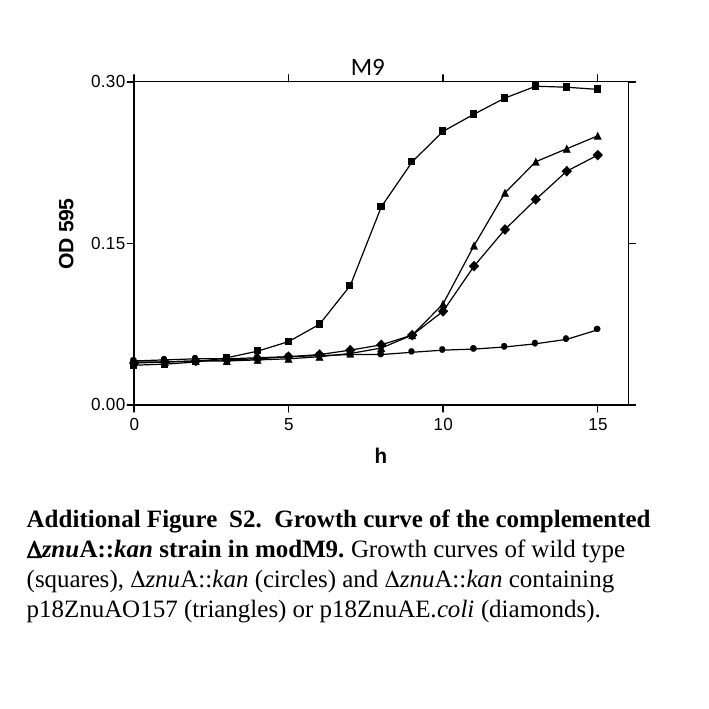

M9
Additional Figure S2. Growth curve of the complemented ΔznuA::kan strain in modM9. Growth curves of wild type (squares), ΔznuA::kan (circles) and ΔznuA::kan containing p18ZnuAO157 (triangles) or p18ZnuAE.coli (diamonds).
