# Supplementary material for: Role of ZnuABC and ZinT in Escherichia coli O157:H7 zinc acquisition and interaction with epithelial cells
Source: BMC Microbiol. 2011 Feb 21;11:36. doi: 10.1186/1471-2180-11-36 (PMC3053223; doi:10.1186/1471-2180-11-36)
Supplement: Additional file 3 — Figure S3: Expression pattern of zinT in SDS-PAGE. The figure shows the total extracellular extracts of zinT::3xFLAG-kan analysed by SDS-PAGE and stained by Coomassie- Blue or revealed by Western blot. [file 1471-2180-11-36-S3.PPTX]

## Slide 1
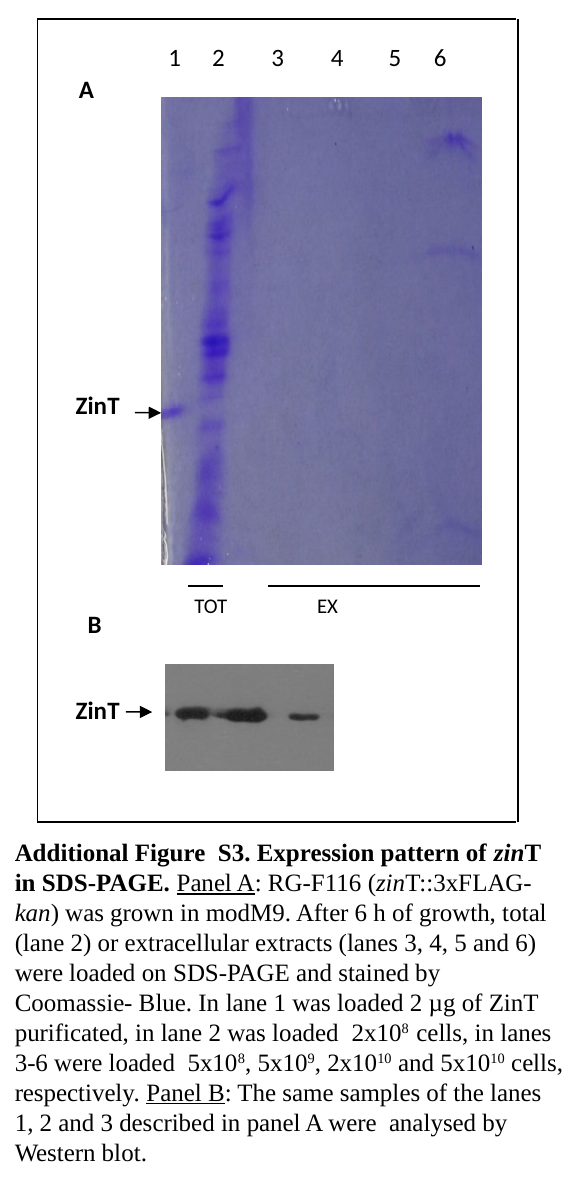

1	2	3	4	5	6
A
ZinT
 TOT EX
B
ZinT
Additional Figure S3. Expression pattern of zinT in SDS-PAGE. Panel A: RG-F116 (zinT::3xFLAG-kan) was grown in modM9. After 6 h of growth, total (lane 2) or extracellular extracts (lanes 3, 4, 5 and 6) were loaded on SDS-PAGE and stained by Coomassie- Blue. In lane 1 was loaded 2 µg of ZinT purificated, in lane 2 was loaded 2x10^8^ cells, in lanes 3-6 were loaded 5x10^8^, 5x10^9^, 2x10^10^ and 5x10^10^ cells, respectively. Panel B: The same samples of the lanes 1, 2 and 3 described in panel A were analysed by Western blot.
