# Supplementary material for: Role of ZnuABC and ZinT in Escherichia coli O157:H7 zinc acquisition and interaction with epithelial cells
Source: BMC Microbiol. 2011 Feb 21;11:36. doi: 10.1186/1471-2180-11-36 (PMC3053223; doi:10.1186/1471-2180-11-36)
Supplement: Additional file 4 — Table S1: Competition assays in CaCo-2 cells. The table shows as during co-infection experiments the znuA mutant strain replicated more efficiently than the wild type strain. [file 1471-2180-11-36-S4.DOC]

**Additional Table S1**

**Competition assays in CaCo-2 cells**

| **Strain A (relevant genotype)** | **Strain B (relevant genotype)** | **Median CI ^a^** | **P ^b^** |
| --- | --- | --- | --- |
|  |  |  |  |
| Wild type | *znuA::cam** | 0.580 | 0.006 |
| Wild type | *znuA::cam** | 0.480 | <0.001 |
|  |  |  |  |

1. Competitive index= output (Strain A/Strain B)/inoculum (Strain A/Strain B).
2. Statistical differences between output and inocula (the P-values) were determined by the Students *t* test.

*Antibiotic used for strains selection
